# Transoral incisionless fundoplication for treatment of gastroesophageal reflux disease in clinical practice

**DOI:** 10.1007/s00464-012-2324-2

**Published:** 2012-05-31

**Authors:** Bart P. L. Witteman, Rob Strijkers, Eva de Vries, Liza Toemen, José M. Conchillo, Wim Hameeteman, Pieter C. Dagnelie, Ger H. Koek, Nicole D. Bouvy

**Affiliations:** 1 Department of General Surgery, Maastricht University Medical Centre, Maastricht, The Netherlands; 2 Department of Gastroenterology, Maastricht University Medical Centre, Maastricht, The Netherlands; 3 Department of Epidemiology, Maastricht University Medical Centre, Maastricht, The Netherlands; 4 Department of Surgery, Maastricht University Medical Centre, P. Debyelaan 25, 6202 AZ Maastricht, The Netherlands

**Keywords:** Endoscopy, Fundoplication, GERD

## Abstract

**Background:**

Transoral incisionless fundoplication is a recently introduced endoluminal technique for the treatment of gastroesophageal reflux disease (GERD). The objective of this study was to determine outcomes in chronic GERD patients who were referred for surgical management.

**Methods:**

A cohort of 38 patients underwent transoral incisionless fundoplication (TIF) in a tertiary care setting. Pre- and post-procedure assessment included GERD-related quality of life questionnaires, proton pump inhibitor (PPI) usage, 24-h pH measurements, upper gastrointestinal endoscopy, esophageal manometry, and registration of adverse events. Duration of follow-up was 36 months.

**Results:**

Gastroesophageal valves were constructed of 4 cm (range, 4–6) in length and 220° (range, 180–240) in circumference. One serious adverse event occurred, consisting of intraluminal bleeding at a fastener site. Hiatal hernia was completely reduced in 56 % and esophagitis was cured in 47 % of patients. Postprocedure esophageal acid exposure did not significantly improve (*p* > 0.05). At 36 (range, 29–41) months follow-up 14 patients (36 %) had undergone revisional laparoscopic fundoplication. Quality of life scores of the remaining cohort showed significant improvement (*p* < 0.0001) and daily use of antisecretory medication was discontinued by 74 %.

**Conclusions:**

Endoluminal fundoplication improved quality of life and reduced the need for PPIs in only a subgroup of patients at 3 years follow-up. The amount of patients requiring additional medication and revisional surgery was high.

Gastroesophageal reflux disease (GERD) is defined as a condition that develops when the reflux of stomach contents causes troublesome symptoms and/or complications and has a prevalence of 10–20 % in western Europe and North America [1]. The goals of clinical management of gastroesophageal disease (GERD) are prompt symptom relief, long-term symptom control, and maintenance of esophageal healing [2, 3]. The current algorithm for the effective treatment of GERD consists of antisecretory medication at all patient care levels [4]. Antireflux surgery is reserved for “refractory“ patients who do not respond symptomatically to a double dose of proton pump inhibitors (PPIs) and those who experience PPI intolerance, complications, or are unwilling to stay on continuous medication lifelong [4–12].

In view of the invasiveness of surgery, a less invasive endoscopic procedure for treatment of GERD would be appealing. Several procedures, based on different mechanisms of action, have been developed [13–16]. Many endoscopic techniques and devices, however, did not withstand the test of randomized controlled trials and many have been withdrawn from the market. One of the latest endoscopic techniques for treatment of GERD is transoral incisionless fundoplication (TIF) [17, 18], which is currently under evaluation. The purpose of this study was to review and report the safety and effectiveness of first-generation technique (TIF1) in 38 chronic GERD patients, who were referred for surgical therapy in our center, a 720-bed tertiary care university hospital.

## Methods

### Patient characteristics

TIF1 was offered to patients who were referred for surgical GERD management, because they required high doses of proton pump inhibitors (PPI) but were refractory, unsatisfied, or unwilling to have a lifelong commitment to medication. Inclusion criteria for the procedure were chronic GERD (>6 months), age 18–75 years, body mass index (BMI) <36 kg m^−2^, and normal or hypotonic lower esophageal sphincter (LES) pressure (<30 mmHg). The presence of gastroesophageal reflux was confirmed by either pathological 24-h esophageal pH monitoring or, in case of the intolerance to the ambulatory 24 h pH-system catheter, upper GI barium radiography in both recumbent and Trendelenburg position following standard protocols. Patients were excluded if they had large hiatal hernia (>5 cm), esophagitis grade C or D in the Los Angeles classification or Barrett’s esophagus [19], hypertonic LES pressure (>30 mmHg), or motility disorders. These criteria were chosen as we assumed that patients who met these criteria would respond well to the new endoscopic fundoplication. Patients underwent follow-up assessment to evaluate treatment effectiveness. Resumption of proton pump inhibitors or revisional standard laparoscopic Nissen fundoplication was offered in case of treatment failure based on relapse of symptoms. Data were collected in a prospective fashion with additional retrospective chart reviews. The research protocol was approved by the Maastricht Medical Ethics Committee (MEC 09-4-046.2/pl).

### Procedure details

TIF1 procedure was performed by using the EsophyX-1™ device (EndoGastric Solutions, Inc., Redmond, WA) under general anesthesia following the TIF1 protocol [20–22]. The device was inserted transorally into the esophagus with the patient in left lateral position. Hiatal hernia, if present, was reduced by pushing the squamocolumnar junction to its natural position below the diaphragm using the built-in vacuum invaginator. The gastroesophageal valve (GEV) was restored with a partial fundoplication using a series of sequential retractions of tissue and placement of multiple polypropylene “serosa-fuse” fasteners circumferentially around the GE junction.

### Safety assessment

The incidence of serious and nonserious adverse events was recorded. Serious adverse events were defined as complications necessitating hospitalization and medical or surgical intervention. Nonserious adverse events represented expected side effects and symptoms.

### Effectiveness assessment

**Fig. 1:**
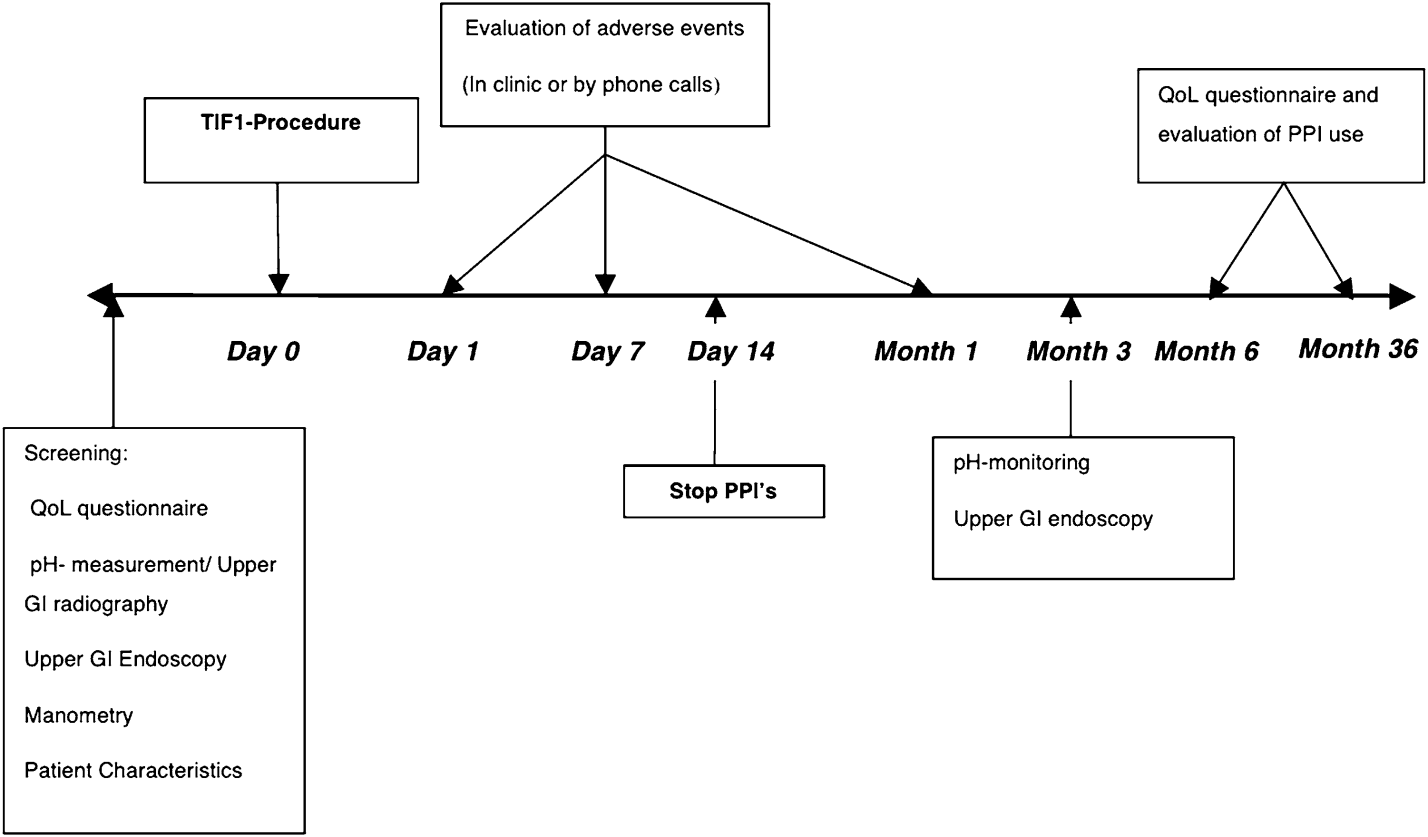
Study timeline

GERD-related quality of life was assessed by the GERD health-related quality of life (GERD-HRQL) questionnaire (see study timeline, Fig. 1) [23–25]. The questionnaire was developed and validated to measure changes of typical GERD symptoms, such as heartburn, in response to surgical or medical treatment. In the present study, an extended version of the questionnaire was used to assess regurgitation. A visual analogue scale ranged from 0 (no symptom) to 5 (worst symptom) and scores ≤2 were indicative of rare or absent symptoms [23, 26]. The heartburn and regurgitation scores were calculated by summing the responses to six questions referring to each symptom, and the scores ≤12 with each score ≤2 were indicative of symptom elimination. Total GERD-HRQL scores ≤30 with each score ≤2 were considered normal. Patients’ satisfaction was evaluated as satisfied, neutral, or dissatisfied [23]. Quality of life was evaluated at baseline while on antisecretory medication and at 6 and 36 months while off medication.

Patients were asked to stop their PPIs at 14 days after the procedure. In case of persisting symptoms, PPIs were resumed on demand and recorded. “Daily” usage of PPIs corresponded to full- or half-dose taken for more than 50 % of the preceding follow-up period, “occasional” to half-dose (or less) taken for <50 % of the preceding follow-up period, and “none” to no medication taken within the specified period.

At 3 months postprocedure, all patients underwent follow-up testing (pH-metry and upper GI endoscopy) while off antisecretory medication. Esophageal acid exposure was measured while off PPIs at screening and at 3 months postprocedure using the Orion II Ambulatory 24-h pH System (Medical Measurement Systems, Enschede, The Netherlands). Normal esophageal acid exposure was defined by pH <4 for ≤4.2 % of the total monitoring time, DeMeester score of <14.72, total number of reflux episodes <50, number of long reflux episodes <4, and the duration of the longest reflux episode <9.2 min. A reduction in esophageal acid exposure to ≤4.2 % of time or by at least 30 % compared with baseline was considered clinically significant [20].

Hiatal hernia and esophagitis were assessed by upper GI endoscopy [27, 28]. The displacement of the squamocolumnar junction proximal to the diaphragmatic hiatus by at least 1 cm was determinant of sliding hiatal hernia and measured endoscopically using standard protocol [29, 30]. The procedure was intended to reduce small- and medium-sized hiatal hernia (1–5 cm). Because hiatal hernia size correlates with the presence and severity of esophagitis, its 50 % reduction was considered clinically significant [31]. Esophagitis was assessed following the Los Angeles classification scale [19], and its reduction by one grade was considered clinically significant.

During upper GI endoscopy, the anatomic aspects of the restored GEVs, were assessed by measuring their body length, defined as the length in cm from the apex of the fundus to the valve lip [32], and circumference between the two most distant fasteners, as well as by estimating adherence to the endoscope (tight, moderate or loose) and Hill grade [33].

### Statistical analysis

Data were analyzed by using SPSS^®^ software version 16.0 (SPSS, Inc., Chicago, IL). Continuous variables, such as age, procedure duration, GERD-HRQL scores, percentage of time at pH <4, DeMeester scores, hiatal hernia size, and valve measurements, were summarized by median and range to better represent the location of the population main trend because of the generally skewed data distribution. Categorical variables, such as adverse events, PPI usage, satisfaction level, Hill grade, adherence, and esophagitis, were summarized as counts and percentages. *P* values for changes at follow-ups compared with baseline within groups were calculated using the Wilcoxon signed-rank test. Predictors of clinical effectiveness were evaluated through correlation analysis between GERD-HRQL scores and anatomic and pathophysiologic variables.

At 36 months follow-up, categorical variables were compared between groups using the Fisher’s exact test and continues variables using Mann–Whitney *U* test. Two-tailed *P* values <0.05 were considered statistically significant. Backward stepwise regression models were used to select independent predictors of failure.

## Results

### Patient characteristics

A total of 38 patients underwent TIF1 procedure between June 2006 and June 2007. The majority of patients were Caucasian males between aged 40 and 50 years and overweight (median BMI, 26.3; range, 20–36). Patients suffered from GERD for median 6 years (range, 1–25), and all were treated with antisecretory medication for median 3 years (range, 1–24). Smoking was reported by 34 % of patients, and 29 % of patients were former smokers. Alcohol was consumed by 57 % of patients at a frequency of either one drink per day (34 %) or 2-4 drinks per day (23 %). PPIs were used daily by 37 (97 %) patients, mostly at a double dose and H2 blockers were used daily by 1 patient. Most patients (95 %) had small-sized (1–2 cm, *n* = 25) and some had medium-sized (3–5 cm, *n* = 11) hiatal hernia, and 39 % of patients had esophagitis (Table 1). The quality of life screening assessment was conducted while patients were taking antisecretory medication and revealed pathologic GERD-HRQL scores in 37 (97 %) patients. Satisfaction index at screening showed 13 (34 %) patients were satisfied, 13 (34 %) were neutral, and 12 (32 %) were dissatisfied with their health condition while using PPIs.

**Table 1:** Patient characteristics at screening

No. patients	38
Female/male	11 (29 %) / 27 (71 %)
Age (year)	46 (22–79)
BMI (kg m^−2^)	26.3 (20.1–36.0)
GERD duration (year)	6 (1–25)
No. on antisecretory medication	38 (100 %)
Duration of PPI use (year)	3 (1–24)
Hiatal hernia	36 (95 %)
Small (1–2 cm)	25 (69 %)
Medium (3–5 cm)	11 (31 %)
Esophagitis	15 (39 %)
Grade A	7 (47 %)
Grade B	8 (53 %)
LES resting pressure (mmHg)	14 (3-27)

Values are medians (ranges) or counts (%)

### Procedure details

Median duration of the procedures was 65 minutes (range, 35–142), and 16 (range, 10–21) fasteners were used to construct the GEV. Most valves measured 4 cm (range, 4–6) in length, 220° (range, 180–240°) in circumference, and were centrally balanced (81 %) and tight (78 %). Hiatal hernia, present in 36 patients, was directly postprocedure reduced to ≤1 cm. Hospitalization stay was 1 day for most (37/38) patients. All were instructed to consume a liquid diet during the first 2 weeks and a soft diet during the following 4 weeks.

### Safety assessment

One serious adverse event occurred and consisted of postoperative bleeding. The night after the procedure, one patient experienced hematemesis and melena. The patient was male, and his hemoglobin dropped from 9.5 to 7.0 mmol/l. Endoscopy was repeated and did not show an active bleeding focus. The patient was closely monitored and remained hemodynamically stable as well as his hemoglobin level. Discharge followed on the third postoperative day, and no further drawbacks were reported at follow-up visits.

In another patient, a mucosal lesion occurred in the esophagus with the introduction of the device. Postoperative upper GI radiography did not show signs of a full thickness perforation, and the event had no further consequences. Other adverse events were mild and resolved spontaneously in the majority of patients within the first week (Table 2). Epigastric pain was most commonly reported by 37 (97 %) patients, further left shoulder pain (29 %), and pharynx irritation (16 %). Dysphagia and gasbloating was reported by four (11 %) patients and lasted up to 1 week.

**Table 2:** Adverse events reported after TIF1 grouped in order of occurrence and by their duration

	Day 1 (%)	Week 1 (%)	Month 1 (%)
Epigastric pain	37	11 (29)	1 (3)
Left shoulder pain	11 (29)	6 (16)	3 (8)
Pharynx irritation	6 (16)	0 (0)	0 (0)
Dysphagia	4 (11)	1 (3)	0 (0)
Fever	4 (11)	2 (5)	0 (0)
Gas bloating	4 (11)	2 (5)	0 (0)
Nausea	4 (11)	0 (0)	0 (0)
Hematemesis	3 (8)	0 (0)	0 (0)
Vomiting	2 (5)	0 (0)	0 (0)
Mucosal tear	1 (3)	1 (3)	0 (0)
Pneumonia	0 (0)	2 (5)	0 (0)

Values represent a percentage of patients experiencing each adverse event at 1 day, 1 week, and 1 month after the procedure

### Clinical effectiveness

At a follow-up period of 6 months (range, 3–15), more than 80 % of patients had normal GERD-HRQL, heartburn, and regurgitation scores (Table 3). Seventy percent of patients were satisfied with their health condition, 27 % were neutral, and 3 % were dissatisfied. Daily use of antisecretory medication was discontinued by 31 (82 %) patients (Table 4) and reduced in dosage by 6 of the remaining 7 patients.

**Table 3:** GERD Health-Related Quality of Life (HRQL), heartburn, and regurgitation scores

	Median (range)	Median % improvement versus baseline on PPIs	*N* (%) normal^a^
GERD-HRQL score			
Pre-TIF1 on PPIs (n = 38)	33 (7–69)	–	–
Post-TIF1 off PPIs	4 (0–51)	87 % (−95 to 100)	31 (82)
6 months (*n* = 38)		*P* < 0.0001	
Post-TIF1 off PPIs	5(0-29)	83 % (−75 to 100)	9 (47)
36 months (*n* = 19)		*P* < 0.0001	
Heartburn score			
Pre-TIF on PPIs (*n* = 38)	17 (5–30)	–	–
Post-TIF off PPIs	3 (0–20)	84 % (−114 to 100)	31 (82)
6 months (*n* = 38)		*P* < 0.0001	
Post-TIF1 off PPIs	0 (0–5)	100 % (−125 to 100)	13 (86)
36 months (*n* = 19)		*P* < 0.0001	
Regurgitation score			
Pre-TIF on PPIs (n = 38)	14 (0–29)	–	–
Post-TIF off PPIs	0 (0–20)	83 % (−200 to 100)	34 (89)
6 months (*n* = 38)		*P* < 0.0001	
Post-TIF1 off PPIs	1(0–5)	90 % (−50 to 100)	13 (86)
36 months (*n* = 19)		*P* < 0.0001	

^a^GERD-HRQL scores were normal if ≤30 and each individual score was ≤2; heartburn and regurgitation scores were normal if ≤12 and each individual score was ≤2

**Table 4:** Usage of proton pump inhibitors before, 6 months after TIF1, and 36 months after TIF1

	Daily (%)	Occasional (%)	None (%)
Baseline	37 (97)	0 (0)	1 (3)
Post-TIF1 6 months (*n* = 38)	7 (18)	6 (16)	25 (66)
Post-TIF1 36 months (*n* = 19)	5 (26)	6 (32)	8 (42)

Values represent counts (%)

Postprocedure esophageal acid exposure while off medication was decreased but did not show significant improvement (Table 5). Hiatal hernia present at baseline was completely reduced in 56 % of patients and reduced in size in 75 % of patients (Table 6). The reduction rate of both small and medium hiatal hernias was equally successful and occurred in 72 and 73 % of patients, respectively. Complete reduction was higher for small hiatal hernia than for medium hiatal hernia size (60 vs. 45 %). At 3 months, esophagitis was not changed significantly and was present in 34 % of patients at postprocedure endoscopic evaluation. Among patients who had esophagitis at baseline, 47 % experienced complete healing and 6 % experienced its reduction by one grade; however, four patients experienced worsening in their scores from endoscopically negative at baseline to grade A (*n* = 1) or grade B (*n* = 3).

**Table 5:** Esophageal pH monitoring while off PPIs pre-TIF procedure and at 3 months follow-up

	Baseline off PPIs (*n* = 36)	Post-TIF1 off PPIs (*n* = 33)	Median % improvement vs. baseline^a^
Percentage time pH <4	7.4 (0.8–54.2)	5.5 (0.6–18.7)	22 % (−875 to 80)
DeMeester score	21 (3–143)	17 (2–63)	20 % (−652 to 82)
No. reflux episodes per 24 h	60 (14–515)	50 (6–131)	23 % (−220 to 71)
No. long (>5 min) episodes per 24 h	2 (0–13)	2 (0–10)	−10 % (−180 to 100)
Longest reflux episode (min)	10 (1–88)	8 (1–45)	17 % (−1,396 to 100)
Patients (%) with normal pH^b^	9 (25 %)	14 (42 %)	–

Values are medians (ranges)

^a^Median % improvement at 3 months was not significant (*P* > 0.05) in all cases

^b^Normal esophageal pH was defined by pH <4 for ≤4.2 % of 24-h monitoring time

**Table 6:** Endoscopic examination of gastroesophageal valves before and 3 months after TIF1

	Baseline (*n* = 38)	3 months post-TIF1 (*n* = 38)
*Hill grade*		
Grade I	3 (8 %)	21 (55 %)
Grade II	11 (29 %)	7 (18 %)
Grade III	12 (32 %)	10 (26 %)
Grade IV	12 (32 %)	0 (0 %)
Reduced versus pre-TIF	–	35/38 (92 %)
*Hiatal hernia*		
Present	36 (95 %)	16 (42 %)
1–2 cm	25 (69 %)	13 (81 %)
3–5 cm	11 (31 %)	3 (19 %)
Reduced >50 %	–	27/36 (75 %)
1–2 cm	–	18/25 (72 %)
3–5 cm		8/11 (73 %)
Eliminated	–	20/36 (56 %)
1–2 cm	–	15/25 (60 %)
3–5 cm	–	5/11 (45 %)
>5 cm	–	0/1 (0 %)
Worsen	–	0/0 (0 %)
*Esophagitis*		
Present	15 (39 %)	13 (34 %)
Grade A	7 (47 %)	6 (46 %)
Grade B	8 (53 %)	7 (54 %)
Grade C+D	0 (0 %)	0 (0 %)
Reduced		8/15 (53 %)
Grade A	–	4/7 (57 %)
Grade B	–	3/8 (38 %)
Grade C+D	–	0/0 (0 %)
Eliminated	–	7/15 (47 %)
Grade A	–	4/7 (57 %)
Grade B	–	3/8 (38 %)
Grade C+D	–	0/0 (0 %)
Worsen		4 (20 %)
None to Grade A		1 (25 %)
None to Grade B		3 (75 %)

At 36 months follow-up, 14 (37 %) patients had requested revisional laparoscopic Nissen fundoplication, because their GERD symptoms were not satisfactory managed. In the revisional surgery, the TIF fundoplication was taken down laparoscopically by dissecting though the serosa-to-serosa fusion layer until anatomy was restored to the pre-TIF situation. Laparoscopic Nissen fundoplication was feasible in all of these patients.

In the remaining cohort of 24 patients, 3 were lost to follow-up and 1 patient was deceased 2 years after the TIF1 procedure of a non-GERD–related cause (ischemic heart disease). In this group of 19 patients, 47 % had normal GERD-HRQL scores and 86 % had normal heartburn and regurgitation scores (Table 3). Seventy percent of patients were satisfied, 27 % were neutral, and 3 % were dissatisfied with their general health condition. Daily use of antisecretory medication was discontinued by 8 (42 %) patients and used occasionally in 32 % (Table 4).

Backward stepwise regression analysis, used to identify patient characteristics that could predict treatment outcomes, showed the presence of esophagitis at screening as the only statistically significant predictor of treatment failure and the demand for revisional laparoscopic fundoplication (Table 7).

**Table 7:** Patient characteristics in relation to the need for revisional laparoscopic fundoplication

No. patients	24	14	*p* value
Female/male	18/6	9/5	0.48
Age (year)	45 (22–79)	51 (25–65)	0.56
BMI (kg m^−2^)	27 (21–36)	26 (20–33)	0.62
GERD duration (year)	5.8 (1–24)	5.8 (1–25)	0.62
Duration of PPI use (year)	3.5 (0.7–24)	2.0 (0.7–15)	0.48
Hiatal hernia			0.45
Small (0–2 cm)	79 %	64 %	
Medium (3–5 cm)	21 %	36 %	
Esophagitis	6/24 (25 %)	9/14 (64 %)	0.04
LES resting pressure (mmHg)	15 (3–27)	12 (4–23)	0.16
3 months post-TIF1 pH-metry	5.5 % (0.6–19)	4.2 % (0.7–14.5)	0.91
total percentage time <4			

Values are medians (ranges) or counts (%)

## Discussion

Medical management of GERD with proton pump inhibitors is effective for 70–80 % of GERD patients; however, the disease is not cured, resulting in a lifelong commitment to drug therapy [34]. Refractory patients seek alternative treatment as well as patients who are unwilling to take lifelong medication, due to the medication costs or suggested side effects, such as osteoporosis, and increased risks on enteric and pulmonary infections [35, 36]. Surgery has been the alternative to drug treatment. The 360° laparoscopic Nissen fundoplication is the current “gold standard,” and partial surgical fundoplication techniques have been shown to be effective for long-term GERD control as well [37].

The invasiveness and side effects, such as dysphagia and gas bloating syndrome, are downsides of current surgical options, and therefore, an effective less invasive endoscopic alternative is appealing [38]. The ideal endoscopic antireflux procedure should be safe, easy to perform, effective, durable and minimally invasive, and have the ability to be performed under conscious sedation [39]. Several endoscopic techniques, based on different mechanisms of action have been tested: tissue remodeling by radiofrequency delivery [14], injection of bulking agents [15], creation of esophageal mucosal tissue pleats by suturing [13], and full-thickness fundoplication [16]. Most of these techniques and devices were not able to control GERD and have been withdrawn from the market.

The TIF procedure was, in contrast to most early endoscopic anti reflux procedures, designed to resemble parts of the surgical fundoplication. The goal is to increase the competence of the antireflux barrier by constructing a full-thickness partial fundoplication by the deployment of transmurally placed polypropylene tissue fasteners in conjunction with circumferential tightening of the distal esophagus. Serosa-to-serosa or serosa-to-muscularis fusion results in the recreation of a 200–300° gastroesophageal valve that can be tailored to the individual patient [20, 22]. The most important difference compared with the surgical approach is the inability of extragastric dissection to reduce (larger) hiatal hernia and to perform cruraplasty.

In the present study, the TIF1 procedure was relatively safe among the 38 patients. We experienced postoperative bleeding in one patient. Another adverse event was an esophageal mucosal tear. After experiencing this event, we started to use olive oil as lubricant for device introduction, which has successfully prevented this complication further in our series. The postoperative dysphagia experienced by 11 % was mild and resolved within 7 days without intervention and could result from edema at the GE junction. Early gas bloating and nausea present in 11 % of patients could be caused by insufflation of the stomach during the procedure, anesthesia, or manipulations of tissue at the GE junction that possibly caused vagal irritation. These mild adverse events also were reported by others as well as postoperative bleeding; however, two full-thickness esophageal perforations occurred upon device insertion in one series [20].

TIF1 technique resulted in a reduction of hiatal hernia and restoring Hill grade I gastroesophageal valves in 75 % of patients, based on endoscopic appearance at the end of the procedure. Complete reduction was higher for small (1–2 cm) hiatal hernia than for medium (3–5 cm) hiatal hernia size, and for this reason we excluded medium hernia size in further studies using this technique. At 6 months follow-up, TIF1 had promising control of their symptoms and quality of life improved in more than 80 % of patients, with cessation of daily GERD medication. Postprocedure PH measurements, however, did not show a significant reduction of distal esophageal acid exposure among the 38 patients. After 3 years, symptoms were not satisfactorily managed among 37 % to such a degree that they requested revisional surgery. Because symptoms worsened over time for these patients after a relatively symptom-free period, durability of the restored GEV using the polypropylene tissue fasteners could be a concern. The “pull-through” of the H-fasteners through the esophageal wall has been suggested as the primary mechanism of failure with this technique [40]. Another explanation of these initial encouraging outcomes could be the placebo effect. In the experience with other endoscopic therapies for GERD, this effect could be as high as 25–50 % [41, 42].

In the remaining cohort of patients, who completed the 3-year follow-up, symptoms were still significantly improved and daily use of antisecretory medication was discontinued by 74 %. Although it remains unclear whether symptoms will increase over time in this group as well. We tried to identify patient characteristics that could predict treatment outcome by the use of backward stepwise regression analysis of our data. Patient characteristics that we recorded in this study were sex, age, BMI, GERD duration, hiatal hernia size, presence of esophagitis at screening, LES resting pressure, and pH measurements. The presence of esophagitis at screening was the only statistically significant predictor of treatment failure with the need for revisional surgery. It is possible that the durability may be worse in these patients, because the diseased tissue in the distal esophagus could possibly cause early slippage of the polypropylene H-fasteners and cause failure of the restored GEV. Although we know from the literature that symptom severity may not be directly related to pathological findings, another possible explanation of failure in this group could be that patients with advanced disease needed revisional surgery earlier compared with patients without esophagitis, after having undergone insufficient treatment.

Early reports on this technique had various outcomes. A feasibility study claimed long-term safety and durability after TIF and showed sustained improvement of symptoms after a 2-year follow-up period in 14 patients. Cessation of PPI therapy was sustained in more than 70 % of patients [43]. In a company-sponsored, multicenter study of 84 patients, complete symptom elimination was achieved in 75 % of patients after 1 year and 85 % were off medication. Lower esophageal acid exposure was reduced in 61 % and normalized in only 37 %. The authors concluded that the procedure was safe and effective for improving subjective and objective outcome measures [20].

Other reports showed less favorable outcomes. In an Italian study, which included 20 patients for the TIF procedure, 4 patients needed laparoscopic fundoplication within the first year. Quality of life was improved in 15 patients at 1-year follow-up, but esophageal acid exposure worsened in 66.7 % of patients versus preprocedure [44].

According to the literature and to this study, the effectiveness of GERD management of endoscopic fundoplication does not compare to the excellent results of laparoscopic antireflux surgery by far [45]. So, will there be a future for endoluminal fundoplication? TIF procedure has undergone technical modifications into the so-called “TIF2 technique” based on a study in canines, which showed superior results in lower esophageal sphincter pressure and length compared with the TIF1 technique and tridimensional vector volume measurements that resembled the Nissen fundoplication [22]. The EsophyX device also has been modified. The shaft of the second generation device is less flexible, which enables the surgeon to apply more torque to the fundoplication and wrap the stomach around the esophagus theoretically more consistent with a surgical fundoplication. Furthermore, we have to consider a learning curve and that more clinical experience with the technique may improve outcomes. Perhaps there will be a group of selected GERD patients who could benefit from the procedure, even if results will never be as good as those from surgery? For instance, patients who are at high risk for surgery due to comorbidities may benefit from the procedure. It also is to be considered that the present study, and other early studies with this new technique, attracted patients who are not satisfied or refractory to PPI treatment. All patients enrolled in this study were referred for surgical fundoplication by their gastroenterologists. Perhaps the TIF-procedure turns out to be much better suitable as initial GERD treatment in selected patients, who also would respond well to PPI treatment? The endoscopic procedure may be an alternative to PPIs as opposed to an alternative to surgical treatment. This would be difficult to investigate, because it may not be easy to enroll patients who are satisfied with their PPI treatment for a study with the new technique.

In the present study, one of the largest, single-center experiences with transoral incisionless fundoplication has been described with the longest follow-up period published. Pre- and postprocedure, subjective as well as objective outcome measures were tested. There are several limitations to this study. We used a nonrandomized design, and there is possible selection bias with the mixed population at inclusion. Another limitation is that we defined failure as the patients’ request for revisional surgical fundoplication based on recurrent symptoms only, without repeating objective measurements in these patients (such as pH measurements and endoscopy) at time of failure.

The TIF1 procedure was relatively safe, and the feared side effects from antireflux surgery—dysphagia and gas bloating—resolved within 1 week postprocedure in this group. Only a subgroup of patients experienced improved quality of life and reduced need for PPIs at 3 years follow-up, and an unacceptably high amount of patients required additional medication or revisional laparoscopic fundoplication. Esophagitis at screening was a predictor for treatment failure of endoscopic fundoplication. Although the endoluminal technique seems attractive because it is minimally invasive and side-effects are mild, according to this study it is not ready for routine GERD treatment. Additional studies are needed to indentify predictors of success and failure more clearly, to explore the technical modifications, and to compare endoluminal fundoplication to conventional treatment modalities for GERD in a randomized study design.

## Abbreviations

GERD: Gastroesophageal reflux disease
TIF: Transoral incisionless fundoplication
PPI: Proton pump inhibitor
